# Supplementary material for: Factors constraining natural recovery of Diadema antillarum following a mass die-off: a case study near the island of Saba, Caribbean Netherlands
Source: PeerJ. 2025 Dec 17;13:e20418. doi: 10.7717/peerj.20418 (PMC12717850; doi:10.7717/peerj.20418)
Supplement: Supplemental Information 1 [file peerj-13-20418-s001.docx]

Table S1: Density (mean ± standard error) of *Diadema antillarum* on four different locations after the 2022 die-off (March 2022) and one year later (March - May 2023).

|  | **Density (n m^-2^)** | |
| --- | --- | --- |
|  | **2022** | **2023** |
| **Diadema City** | 0.03 ± 0.01 | 1.43 ± 0.51 |
| **Tent Reef** | 0.03 ± 0.02 | 0.02 ± 0.01 |
| **Ladder Bay** | 0.00 ± 0.00 | 0.00 ± 0.00 |
| **Torrens Point** | 0.01 ± 0.01 | 0.00 ± 0.00 |

Table S2: Average predation pressure on *D. antillarum* (gr 100m^-2^, ± SE) for seven species with the highest predation pressure, six other encountered species, and in total per location. Species were sorted based on their overall predation pressure.

| **Average predation pressure (gr per** **100m^-2^, ± SE)** | | | | | |
| --- | --- | --- | --- | --- | --- |
| **Common name** | **Scientific name** | **Diadema City** | **Tent Reef** | **Ladder Bay** | **Torrens Point** |
| Caesar grunt | *H. carbonarium* | 15.1 ± 7.9 | 0.0 ± 0.0 | 22.7 ± 12.6 | 73.3 ± 40.8 |
| Black Margate | *A. surinamensis* | 0.0 ± 0.0 | 0.0 ± 0.0 | 81 ± 80.4 | 29.7 ± 29.7 |
| Spanish hogfish | *B. rufus* | 0.0 ± 0.0 | 84.1 ± 39.2 | 18.1 ± 9.1 | 3.3 ± 2.4 |
| Puddingwife | *H. radiatus* | 0.2 ± 0.2 | 18.9 ± 18.9 | 1.0 ± 1.0 | 29.2 ± 29.2 |
| Bluehead | *T. bifasciatum* | 0.7 ± 0.2 | 13.7 ± 4.4 | 5.0 ± 1.4 | 2.3 ± 0.5 |
| Yellowhead wrasse | *H. garnoti* | 1.4 ± 0.8 | 0.7 ± 0.5 | 6.5 ± 2.2 | 2.3 ± 1.6 |
| French grunt | *H. flavolineatum* | 6.5 ± 1.8 | 0.0 ± 0.0 | 0.0 ± 0.0 | 2.2 ± 1.5 |
| 6 other species |  | 1.1 ± 1.1 | 2.0 ± 1.1 | 1.1 ± 1.1 | 0.3 ± 0.1 |
| **Total** |  | **25.0 ± 8.9** | **119.4 ± 39.7** | **135.4 ± 93.4** | **142.6 ± 72.5** |

Table S3: *D. antillarum* predator abundance (ind survey^-1^, ± SE) and predation pressure on *D. antillarum* (gr survey^-1^, ± SE) per location and in total, recorded during the 20 minute roving diver surveys. Species were sorted based on their overall abundance.

| **Average abundance of fish predators (ind survey^-1^, ± SE)** | | | | | |
| --- | --- | --- | --- | --- | --- |
| **Common name** | **Scientific name** | **Diadema City** | **Tent Reef** | **Ladder Bay** | **Torrens Point** |
| Caesar grunt | *H. carbonarium* | 12.7 ± 2.2 | 2.3 ± 0.7 | 11 ± 3.5 | 10.7 ± 4.2 |
| Spanish hogfish | *B. Rufus* | 1.3 ± 0.3 | 6.3 ± 1.3 | 10 ± 2.9 | 8.3 ± 2.3 |
| French grunt | *H. flavolineatum* | 11.3 ± 2.3 | 1.3 ± 0.9 | 7 ± 1.7 | 3.3 ± 1.5 |
| Black margate | *A. surinamensis* | 10 ± 1 | 0.3 ± 0.3 | 1.3 ± 0.7 | 1 ± 0.6 |
| Smooth trunkfish | *L. triqueter* | 3.7 ± 2.3 | 2.7 ± 0.3 | 0.7 ± 0.7 | 1.3 ± 0.7 |
| Spotted trunkfish | *L. bicaudalis* | 1.3 ± 0.9 | 0.0 ± 0.0 | 0.3 ± 0.3 | 0.0 ± 0.0 |
| Puddingwife | *H. radiatus* | 0.0 ± 0.0 | 0.3 ± 0.3 | 0.7 ± 0.3 | 0.3 ± 0.3 |
| Porcupinefish | *D. hystrix* | 0.7 ± 0.3 | 0.0 ± 0.0 | 0.0 ± 0.0 | 0.0 ± 0.0 |
| Sharpnose puffer | *C. rostrata* | 0.0 ± 0.0 | 0.0 ± 0.0 | 0.0 ± 0.0 | 0.3 ± 0.3 |
| **Total** |  | **41 ± 4.7** | **13.2 ± 1.9** | **31 ± 3.6** | **25.2 ± 3.7** |
| **Average predation pressure (gr survey^-1^, ± SE)** | | | |  |  |
| **Common name** | **Scientific name** | **Diadema City** | **Tent Reef** | **Ladder Bay** | **Torrens Point** |
| Caesar grunt | *H. carbonarium* | 406 ± 113 | 62 ± 27 | 281 ± 75 | 183 ± 60 |
| Spanish hogfish | *B. Rufus* | 27 ± 15 | 211 ± 49 | 327 ± 88 | 246 ± 106 |
| French grunt | *H. flavolineatum* | 22 ± 5 | 1 ± 1 | 9 ± 4 | 5 ± 2 |
| Black margate | *A. surinamensis* | 1753 ± 310 | 54 ± 54 | 434 ± 250 | 274 ± 158 |
| Smooth trunkfish | *L. triqueter* | 10 ± 6 | 9 ± 3 | 2 ± 2 | 2 ± 2 |
| Spotted trunkfish | *L. bicaudalis* | 39 ± 20 | 0 ± 0 | 5 ± 5 | 0 ± 0 |
| Puddingwife | *H. radiatus* | 0 ± 0 | 53 ± 53 | 55 ± 30 | 20 ± 20 |
| Porcupinefish | *D. hystrix* | 339 ± 169 | 0 ± 0 | 0 ± 0 | 0 ± 0 |
| Sharpnose puffer | *C. rostrata* | 0 ± 0 | 0 ± 0 | 0 ± 0 | 0 ± 0 |
| **Total** |  | **2596 ± 498** | **390 ± 175** | **1113 ± 256** | **730 ± 154** |

Table S4: Abundance of *D. antillarum* micro and macro invertebrate predators (ind 100m^-2^, ± SE) per location and in total. Invertebrate groups were sorted based on their overall abundance.

| **Average abundance of micro predators (ind 100m^-2^, ± SE)** | | | | | |
| --- | --- | --- | --- | --- | --- |
| **Common name** | **Scientific name or infraorder** | **Diadema City** | **Tent Reef** | **Ladder Bay** | **Torrens Point** |
| Shrimp | Caridea | 899.8 ± 147.5 | 56.1 ± 12.3 | 27.2 ± 10 | 22.6 ± 8.9 |
| Hermit crab | Anomura | 1.1 ± 0.7 | 0.7 ± 0.3 | 2.6 ± 0.7 | 1.1 ± 0.5 |
| Crab other | Brachyura | 0.6 ± 0.3 | 2.0 ± 0.8 | 0.4 ± 0.2 | 0.4 ± 0.2 |
| Spotted lobster | *P. guttatus* | 0.0 ± 0.0 | 0.0 ± 0.0 | 0.0 ± 0.0 | 0.2 ± 0.2 |
| King helmet | *C. madagascariensis* | 0.0 ± 0.0 | 0.2 ± 0.2 | 0.0 ± 0.0 | 0.0 ± 0.0 |
| **Total** |  | **901.7 ± 147.1** | **59.1 ± 12.2** | **30.9 ± 10.0** | **24.4 ± 9.1** |
| **Average abundance of macro predators (ind 100m^-2^, ± SE)** | | | | | |
| **Common name** | **Scientific name or infraorder** | **Diadema City** | **Tent Reef** | **Ladder Bay** | **Torrens Point** |
| Spotted spiny lobster | *P. guttatus* | 1.5 ± 0.6 | 0.7 ± 0.3 | 0.0 ± 0.0 | 0.6 ± 0.3 |
| Hermit crab | Anomura | 0.9 ± 0.5 | 0.0 ± 0.0 | 0.7 ± 0.3 | 0.7 ± 0.4 |
| Caribbean spiny lobster | *P. argus* | 0.2 ± 0.2 | 0.2 ± 0.2 | 0.0 ± 0.0 | 0.0 ± 0.0 |
| King helmet | *C. madagascariensis* | 0.0 ± 0.0 | 0.0 ± 0.0 | 0.2 ± 0.2 | 0.0 ± 0.0 |
| **Total** |  | **2.6 ± 0.7** | **0.9 ± 0.3** | **0.9 ± 0.3** | **1.3 ± 0.4** |
